# Lecture on the Treatment of Catarrh and Bronchitis

**Published:** 1870-03

**Authors:** George Johnson

**Affiliations:** Prof. Medicine in King’s College; Physician to King’s College Hospital


### LECTURE ON THE TREATMENT OF CATARRH AND BRONCHITIS.

By GEORGE JOHNSON, M.D., F.R.C.P.; Prof. Medicine in King's College; Physician to King's College Hospital.

An ordinary catarrh, although not a dangerous or a very serious disease, is yet, with many persons, an oft-recurring malady, which occasions a great amount of discomfort and annoyance both to the patient and to his associates; and, as treatment has considerable influence upon the progress of the disorder, it is worth while to give the subject careful consideration.

The exciting cause of a catarrh, in the great majority of cases, is a chill, or some unknown atmospheric influence, which tends to suppress the action of the skin; and the most successful plan of treatment consists in the employment of means for restoring the free action of the skin. The popular domestic treatment consists in the use of a hot foot-bath, at bedtime, a fire in the bedroom, a warm bed, and some hot drink taken after getting into bed, the diaphoretic action being assisted by an extra amount of bed-clothes. Complete immersion in a warm bath is more efficacious than a foot-bath; but the free action of the skin is much more certainly obtained by the influence of hot air—most surely and profusely, perhaps, by the Turkish bath. The Turkish bath, however, is not always to be had, and even when available, its use in the treatment of catarrh is attended with some inconvenience. In particular, there is the risk of a too speedy check to the perspiration, after the patient leaves the bath. On the whole, the plan which combines in the greatest degree efficiency with universal applicability consists in the use of a simple hot-air bath, which the patient can have in his own bedroom. All that is required is a spirit-lamp with a sufficiently large wick. Such lamps are made of tin, and sold by most surgical instrument makers.

The lamp should hold sufficient spirit to burn for half an hour. The patient sits undressed in a chair with the lamp between his feet, rather than under the chair. An attendant then takes two or three blankets and folds them round the patient from his neck to the floor, so as to inclose him and the lamp, the hot air from which passes freely round his body. In from a quarter to half an hour there is usually a free perspiration, which may be kept up for a time by getting into bed between hot blankets. I have myself gone into a hot-air bath suffering from headache, pain in the limbs, and other indications of a severe incipient catarrh, and in the course of half an hour I have been entirely and permanently freed from these symptoms by the action of the bath.

Another simple and efficient mode of exciting the action of the skin, consists in wrapping the undressed patient in a sheet wrung out of warm water, then, over this, folding two or three blankets. The patient may remain thus "packed" for an hour or two, until free perspiration has been excited.

I may mention, in passing, that the hot-air bath and the wet packing are very useful in the treatment of many forms of disease. I constantly employ both in the treatment of renal disease; and not long since, I believe that by the wet packing, I saved the life of a lady in whom very alarming symptoms were associated with the imperfect outcoming of the rash of scarlatina.

Now, to return to the treatment of catarrh, let me impress upon you that the sweating plan of treatment, to be successful in cutting short the disease, must be adopted early—I mean within a few hours from the commencement of the symptoms.

Another mode of treating catarrh, which is very successful with patients who are tolerant of opium, consists in giving a dose of opium, or morphia, at bedtime. Within half an hour after the opiate is taken, it frequently happens that the unpleasant coryza, and every other symptom of catarrh, have passed away. If the patient can avoid exposure on the following day, the cure may be complete, and there is no need to repeat the dose.

It is probable that the good effect of the opiate is partly due to its diaphoretic action, which may be increased by combining it with ipecacuanha; but besides its action upon the skin, there must be some direct influence on the nerves and vessels of the inflamed mucous membrane, to explain the speedy relief from discomfort which follows the opiate dose. The opiate treatment of catarrh is not so generally applicable as the sweating plan, for the reason that many persons are intolerant of opium, or they cannot take it without suffering from headache, nausea, and other distressing symptoms, which render it an undesirable remedy for them. In any case, the opiate treatment, like the diaphoretic method, is more successful in proportion as it is resorted to early in the attack.

In some persons, repeated doses of ammonia have the effect of lessening the coryza and other distressing catarrhal symptoms. Five grains of sesquicarborate of ammonia, or a drachm of the aromatic spirit, may be taken in water every three hours. A single dose of ammonia at bedtime is an efficient and useful diaphoretic, its action being aided by external warmth. Some catarrhal patients experience great relief from an occasional dose of spirit of camphor. The usual dose is from 10 to 30 drops in a wineglassful of water. In ordinary catarrh, as a rule, no change of diet is required. A catarrh which has gone on unchecked for a few days, is sometimes much mitigated by a generous diet.

Those who are especially liable to catarrh should be careful to keep their feet warm and dry; and they should be warmly clothed, wearing woolen next the skin. They should avoid excessive wrapping up, since this, with even gentle exercise, tends to overheat the body, and so to increase the risk of a subsequent chill The practice of wearing a hare-skin, wash-leather, or thick folds of flannel over the chest, is to be condemned as at once filthy and unwholesome.

It may be well to remind catarrhal subjects that the nose is a natural respirator, so that in passing from a hot room into the open air, if the mouth be kept closed, the air, in its passage through the nostrils, has its temperature raised before it enters the chest.

There is reason to believe that the daily use of a cold sponge-bath, or a shower-bath, has a wholesome hardening influence upon those who adopt it, and that it renders them less liable to attacks of catarrh.

Treatment of Acute Bronchitis.—Acute bronchitis is an exaggerated catarrh; the two diseases are essentially the same, and they require the same principle of treatment, only modified according to the character of the symptoms.

In the early stage of acute bronchitis, when the mucous membrane is dry and swollen, the hot-air bath or the wet packing may be employed once, or oftener, with advantage. Another very useful remedy in this stage is tartar emetic, in doses of one-sixth of a grain, combined with liquor ammoniæ acetatis. This mixture exerts a diaphoretic action both upon the skin and the mucous membrane of the air-passages; thus it brings on the stage of secretion, and with this a mitigation of the vascular engorgement, The patient should remain in bed, and the temperature of the room should be maintained at from 60 deg. to 65 deg., the air being kept moist by the steam from the spout of a kettle, or a special boiler on the fire. The inhalation of steam, repeated several times in the course of the day, is often very soothing and beneficial. Hot fomentations may be applied to the front and back of the chest by means of spongio-piline, or flannels covered with Mackintosh. A mild mustard-poultice to the front of the chest is a good remedy for a sense of tightness and dyspnœa; but I advise you not to excite painful inflammation of the skin by mustard or turpentine, or by any other means.

When dyspnœa, with a feeling of tightness and oppression at the chest, is urgent and distressing, the application of a few leeches to the chest, or a moderate abstraction of blood by cupping, often affords prompt, decisive, and permanent relief. Venesection is very rarely required; though in the case of a plethoric subject suddenly seized with general capillary bronchitis, and threatened with death from apnœa, venesection may prove a life-saving remedy Milk and beef-tea form the most suitable diet during this stage of the disease. Stimulants and opiates are to be avoided, as a rule, on account of their tendency to increase the congestion and dryness of the inflamed mucous membrane. In the second stage, when a free secretion has been established, antimony and acetate of ammonia are to be discontinued. At this period, a combination of sesquicarbonate of ammonia, with spirit of chloroform, is useful as a stimulating expectorant and anti-spasmodic. Brandy or wine in moderate quantities may now be required to sustain the strength. When, in the advanced stages, there is a profuse purulent secretion, with copious perspirations, the ammonia mixture may be replaced by one, each dose of which contains a grain of sulphate of quinia, two grains of sulphate of zinc, and twenty minims of aromatic sulphuric acid. This combination often checks very rapidly the excessive secretion from the bronchial mucous membrane. The stimulating expectorants are sometimes useful at this stage of the disease—I mean senega, squills, ammoniacum, and the compound tincture of benzoin. If, as sometimes happens, the stimulating expectorants suddenly che[illegible]k secretion, tighten the breath, and increase dy pnœa, their employment must at once be discontinued. When the secretions accumulate and threaten suffocation, the patient being blue, and cold, and drowsy, and the cough nearly or quite ceasing, an emetic of sulphate of zinc is often wonderfully efficacious in clearing the air-passages.

Here I must give you an especial warning in regard to opium. A patient who had been sitting up in bed, laboring for breath day and night, naturally craves for sleep, and begs for an opiate. Now, a small dose of opium given in such a case, has caused fatal narcotism in numberless instances. The opiate stops the cough, and, of course, the expectoration; the patient sleeps more and more heavily; meanwhile the secretion accumulates, and causes fatal apnœa. Never, therefore, give an opiate to a bronchitic patient who has the slightest blueness of the lips. When the expectoration is quite free and the lips are florid, you may sometimes venture to give a small opiate, with antimony or ipecacuanha, or you may give a drachm of the compound tincture of camphor, or twenty minims of chlorodyne. The good effect of a few hours' sleep thus procured are sometimes very manifest.

When bronchitis is associated with blood-contamination, consequent on Bright's disease, diaphoretics, purgatives, and dry cupping over the loins, are amongst the most useful remedies.

The treatment of CHRONIC BRONCHITIS is essentially the same as that of the acute form of the disease. They merge into each other by imperceptible degrees. An acute attack may subside into a chronic condition, and exposure to cold will quickly convert chronic into acute bronchitis.

Amongst other remedies in the chronic stage, the inhalation of the vapor of creastoe, or oil of turpentine, by means of Nelson's inhaler, is often beneficial. These vapors facilitate expectoration at the same time that they tend to check the profuse purulent secretion. The abundant secretion may sometimes be checked by inhaling, in the form of spray, a solution of tanic acid.

In treating diseases of the air-passages by the inhalation of vapors, bear in mind that these vapors rapidly pass beyond the lungs: they are quickly absorbed and enter the circulation, causing, in some instances, headache and other discomforts. The necessary contamination of the blood by the inhalation of vapors renders this mode of medication less generally useful than it otherwise might be in the treatment of bronchial inflammation and catarrh.

Change of air, and, in particular, a residence in a mild, dry, and equable climate, are amongst the most important remedial and preventive measures.—Brit. Med. Jour., Oct., 23, 1869.—Med. News.

